# Development of Mannitol-Based Microparticles for Dry Powder Inhalers: Enhancing Pulmonary Delivery of NSAIDs

**DOI:** 10.3390/ph18060923

**Published:** 2025-06-19

**Authors:** Petra Party, Zsófia Ilona Piszman, Rita Ambrus

**Affiliations:** Institute of Pharmaceutical Technology and Regulatory Affairs, University of Szeged, Eötvös Street 6, 6720 Szeged, Hungary; party.petra@szte.hu (P.P.); piszman.zsofi@gmail.com (Z.I.P.)

**Keywords:** dry powder inhaler, mannitol, meloxicam, meloxicam-potassium, wet milling, spray drying, Andersen Cascade Impactor

## Abstract

**Background/Objectives:** Chronic lung diseases are among the leading causes of death worldwide. In the treatment of these diseases, non-steroidal anti-inflammatory drugs can be effective. We have previously developed an excipient formulation alongside a modern manufacturing protocol, which we aim to further investigate. We have chosen two new model drugs, meloxicam (MX) and its water-soluble salt, meloxicam-potassium (MXP). The particles in dry powder inhaler (DPI) formulation were expected to have a spherical shape, fast drug release, and good aerodynamic properties. **Methods:** The excipients were poloxamer-188, mannitol, and leucine. The samples were prepared by spray drying, preceded by solution preparation and wet grinding. Particle size was determined by laser diffraction, shape by scanning electron microscopy (SEM), crystallinity by powder X-ray diffraction (PXRD), interactions by Fourier-transform infrared spectroscopy (FT-IR), in vitro drug dissolution by paddle apparatus, and in vitro aerodynamic properties by Andersen cascade impactor and Spraytec^®^ device. **Results:** We achieved the proper particle size (<5 μm) and spherical shape according to laser diffraction and SEM. The XRPD showed partial amorphization. FT-IR revealed no interaction between the materials. During the in vitro dissolution tests, more than 90% of MX and MXP were released within the first 5 min. The best products exhibited an aerodynamic diameter of around 4 µm, a fine particle fraction around 50%, and an emitted fraction over 95%. The analysis by Spraytec^®^ supported the suitability for lung targeting. **Conclusions:** The developed preparation process and excipient system can be applied in the development of different drugs containing DPIs.

## 1. Introduction

Every year more than 18 million deaths are caused by respiratory-related diseases. In 2021 this accounted for 27.4% of total deaths worldwide [1]. Therefore, these diseases constitute a significant public health burden worldwide, with current statistics indicating that conditions such as chronic obstructive pulmonary disease (COPD), asthma, and pulmonary infections are prevalent and often interrelated. Additionally, the COVID-19 pandemic has significantly illustrated the vulnerability of respiratory systems, leading to a rise in acute respiratory distress syndrome (ARDS), particularly among individuals with pre-existing respiratory conditions [2,3].

Conventional treatment options for respiratory diseases primarily involve pharmacological interventions, respiratory therapies, and supportive care strategies. Pharmacological treatment typically encompasses bronchodilators, inhaled corticosteroids, and anticholinergics to alleviate symptoms and reduce exacerbation frequencies. Unfortunately, they are effective in reducing symptoms, but do not target the disease’s root causes. For example, glucocorticoids have been shown to effectively manage inflammation and prevent acute exacerbations in pediatric populations [4]. However, these treatments may produce side effects such as oral thrush, suppression of systemic hormones, or an increased risk of pneumonia, necessitating careful management [5]. Moreover, adjunct therapies, including pulmonary rehabilitation, have been pivotal in improving patient outcomes, even though their application remains limited by individual patient circumstances [6,7]. Recent studies highlight the potential of biologics and targeted therapies in managing chronic respiratory diseases. Biologics are emerging as a promising avenue, particularly for asthma and severe COPD, but their integration into treatment models is still developing [8]. Treatment landscape for respiratory diseases is expanding due to ongoing research into novel therapies and innovative drug delivery systems [9]. Personalized medicine and biological therapies represent the forefront of treatment strategies, aiming to address the complexities of respiratory pathologies while enhancing patient outcomes [10].

For the treatment of respiratory-related diseases, targeted delivery directly into the lungs can be beneficial since it has excellent bioavailability and avoids first-pass clearance in the liver [11,12,13]. Due to this bypass of the gastrointestinal and hepatic systems, considerably lower doses are typically required when a drug is delivered via the pulmonary route compared to oral administration. This dose-sparing effect not only reduces the potential for side effects but also decreases the likelihood of dose-dependent toxicity [14,15,16].

Dry powder inhalers (DPIs) represent a promising and increasingly utilized option for pulmonary drug delivery. One of the key benefits of DPIs is their propellant-free operation. Unlike pressurized metered-dose inhalers, which rely on hydrofluoroalkane propellants, DPIs use their own inspiratory effort of the patients to disperse and deliver the medication. This makes them a more environmentally sustainable choice. In addition to their ecological advantages, DPIs are compact, portable, and easy to use, which increases patient adherence. The solid-state nature of DPI formulations also offers significant formulation and storage benefits. Compared to liquid-based systems, powders exhibit enhanced physicochemical stability, which contributes to prolonged shelf-life and reduced need for cold chain logistics. Moreover, the solid form allows for more versatile formulation strategies, including the incorporation of poorly water-soluble drugs, nanoparticles, and controlled-release carriers [17,18,19,20,21].

The physicochemical properties of the final DPI formulation are heavily influenced by the selection and ratio of excipients used during production. The careful optimization of their ratio is essential to balancing stability with aerodynamic efficiency and ensuring that the final product meets the rigorous demands of pulmonary drug delivery systems [22]. Common excipients that stabilize formulations during spray drying and guarantee product stability are mannitol and leucine [23]. Mannitol, a sugar alcohol, is well-known for its stabilizing capacity during the drying process, as it helps protect the drug from thermal and shear stresses that can lead to degradation or loss of activity. As a cryoprotectant, mannitol also preserves the structural integrity of particles during processing and lyophilization steps, thereby contributing to the overall physical stability of the formulation. An additional benefit of mannitol is its ability to promote the formation of spherical particles, which are generally associated with improved flow properties and more consistent aerodynamic behavior. However, one limitation of mannitol is its tendency to crystallize over time. This crystallization behavior can potentially compromise aerosol performance by altering particle size distribution and surface properties during storage, leading to reduced fine particle delivery efficiency [24,25]. Leucine is an amino acid widely used in DPI formulations. Its primary contribution lies in improving the aerosolization properties of the powder. Leucine has a high tendency to migrate to the particle surface during spray drying, forming a hydrophobic shell that reduces particle–particle interactions and enhances dispersibility. This surface enrichment minimizes cohesion and adhesion forces, ultimately resulting in better powder flow and deagglomeration during actuation. Furthermore, leucine has been shown to function as a dispersibility enhancer by decreasing the cohesive forces between particles, thereby lowering the energy required for powder dispersion upon inhalation. This is particularly valuable for achieving high fine particle fractions and consistent dose delivery, which are critical quality attributes for effective pulmonary therapy [26,27,28,29,30].

In this work, our model’s active pharmaceutical ingredients (APIs) were meloxicam (MX) and meloxicam-potassium (MXP), which are non-steroid anti-inflammatory drugs [31,32]. It is commercially available, mainly indicated for arthritis, with oral delivery. It is a great candidate for repositioning, with an alternative route of administration, such as pulmonary delivery. Pharmacological studies demonstrated its antioxidant, antifibrotic, and analgesic activity, so it can be advantageous for patients with cystic fibrosis, chronic obstructive pulmonary disease, lung cancer, or lung inflammation [33,34,35,36,37,38]. Therefore, lung delivery of MX can be advantageous for its systemic anti-inflammatory effect and for local applications as well. However, there are no meloxicam-containing products available on the market for administration by the inhaled route. One of the disadvantages of MX is its poor water solubility, which is the reason for the application of the second model drug. MXP has the same effect as MX, but with better solubility. This novel salt form of MX was developed by Egis Plc. (Budapest, Hungary) and is a valuable intermediate for the synthesis of high-purity MX [39]. There are available publications regarding the pulmonary application of MX and MXK [40,41,42,43,44,45].

Previously, we successfully developed two carrier-free DPI formulations, one of which contains a combination of ibuprofen and mannitol, and one with only mannitol as its API [46]. During the development, we established an effective production procedure, but most importantly, we also created an innovative excipient composition and proved its suitability for pulmonary delivery. The innovative excipient system consists of Poloxamer 188 (POL), used as an excipient to stabilize the microparticles, leucine (LEU), and mannitol (MAN). To accurately assess the impact of the excipient system on the properties of the finished DPI products, we used the same materials in the same ratio, except we changed the active ingredient from the previously used ibuprofen to two new model drugs. Our main goal was to evaluate the innovative excipient system with the new model APIs; therefore, we developed formulations containing MX and ones with MXP. We expected the finished DPI formulations to have spherically shaped, micro-sized particles, rapid active ingredient release, and suitable aerodynamic properties.

## 2. Results

### 2.1. Result of Particle Size Analysis by Laser Diffraction

The initial diameter of the API (D[0.5] = 9.91 ± 0.37 μm) was successfully reduced to 1.81 ± 0.09 μm by wet milling. In the case of MX-containing samples, the D[0.5] values of the samples were between 3 and 5 μm following spray drying (Table 1). One of the most crucial elements in the lung deposition point is the particle size [47]. Only in the case of MXP_MAN_LEU1_SPD from the MXP-containing samples did the outcome meet the standards of the pulmonary powders. Only the D[0.5] values of MX_MAN_LEU0.5_SPD and MXP_MAN_LEU0.5_SPD showed a significant difference when the two sample groups were compared (*p* < 0.001). According to the Span values, the particle size distribution for MX_MAN_LEU0.5_SPD and MX_MAN_LEU1_SPD was monodisperse (Span 2.0). This aids in proper dose calculation and deposition. When the two different API-containing samples were compared, significant difference was detected only between the Span values of MX_MAN_LEU0.5_SPD and MXP_MAN_LEU0.5_SPD (*p* < 0.05).

### 2.2. Outcomes of Density and Powder Flow Tests

Low density DPIs may result in an improved airway deposition profile. The tapped density (ρ_t_) of the samples was approximately 0.3 g/cm^3^ or less (Table 2). The samples can be regarded as low-density formulations because the commercially available DPIs have a density of about 1 g/cm^3^. Furthermore, the findings are promising, due to the fine particle fraction (FPF) increasing as the ρ_t_ decreases. Hausner ratio (HR) and Carr index (CI) values can be used to determine the powder flow, which has an additional impact on aerosolization. MX_MAN_LEU0.5_SPD showed the best results for proper aerodynamic performance.

### 2.3. Findings of Scanning Electron Microscopy Investigation

**Figure 1:**
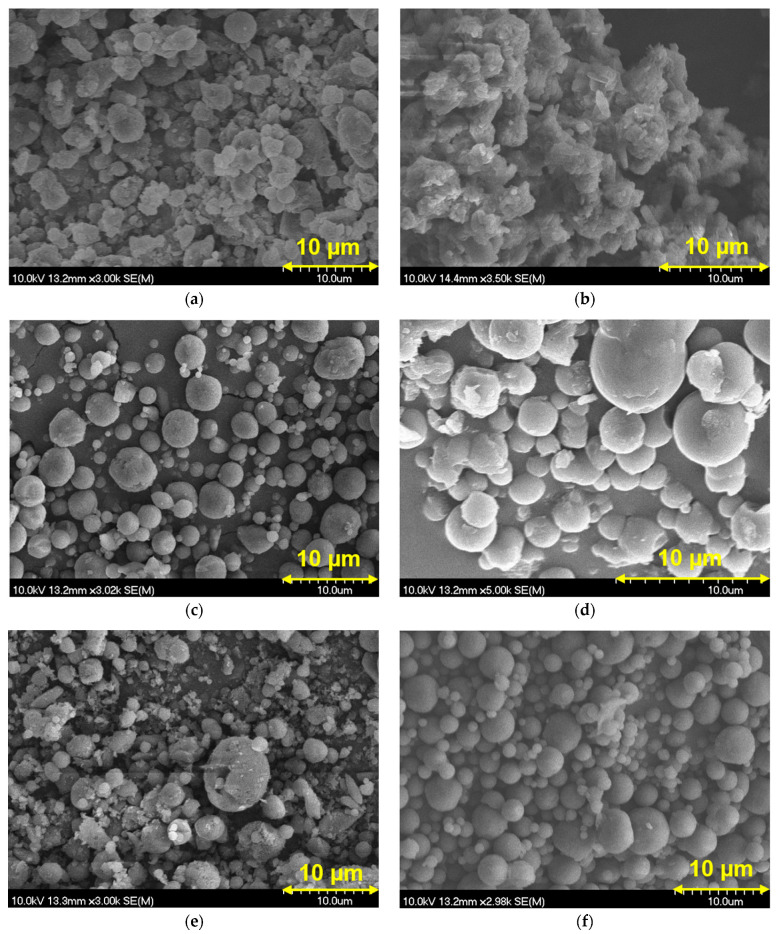
SEM pictures of SPD samples: (**a**) MX_MAN_LEU0_SPD, (**b**) MXP_MAN_LEU0_SPD, (**c**) MX_MAN_LEU0.5_SPD, (**d**) MXP_MAN_LEU0.5_SPD, (**e**) MX_MAN_LEU1_SPD, (**f**) MXP_MAN_LEU1_SPD.

The morphological analysis of the samples (Figure 1) revealed that the particles produced in formulations containing both MAN and LEU exhibited a predominantly spherical shape, which is generally considered advantageous for inhalable powders, as it contributes to improved flowability and reduced agglomeration. In the absence of LEU, the samples showed a tendency toward particle aggregation. This can be attributed to increased cohesive forces and surface energy among the particles, which compromise dispersibility. The presence of LEU significantly improved the powder morphology by mitigating particle–particle interactions. LEU forms a thin, low-surface-energy shell around each particle, which reduces adhesion and cohesion, thereby minimizing aggregation and enhancing powder flow. The observed well-separated spherical particles are more likely to ensure effective delivery to the respiratory tract.

### 2.4. Results of Structure Analysis Using Powder X-Ray Diffraction

**Figure 2:**
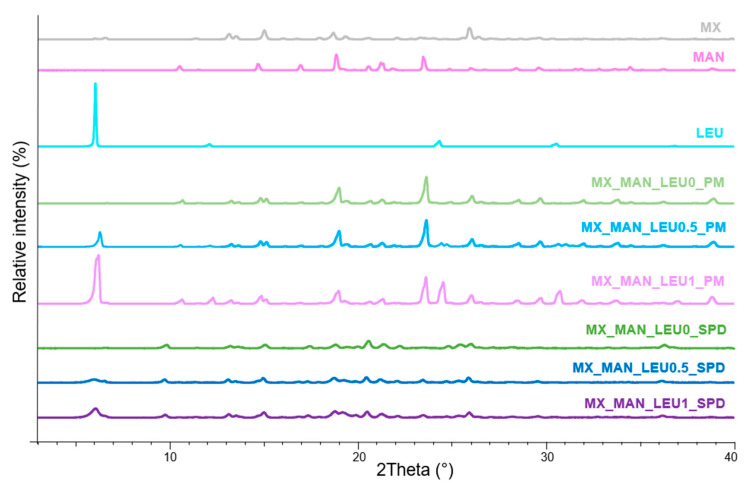
XRPD results of raw materials (MX, MAN, LEU), PMs (MX_MAN_LEU0_PM, MX_MAN_LEU0.5_PM, MX_MAN_LEU1_PM), and SPD samples (MX_MAN_LEU0_SPD, MX_MAN_LEU0.5_SPD, MX_MAN_LEU1_SPD).

**Figure 3:**
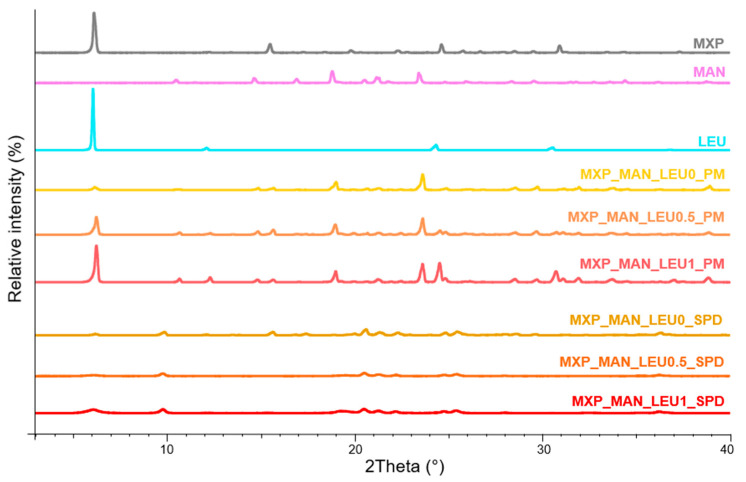
XRPD results of raw materials (MXP, MAN, LEU), PMs (MXP_MAN_LEU0_PM, MXP_MAN_LEU0.5_PM, MXP_MAN_LEU1_PM), and SPD samples (MXP_MAN_LEU0_SPD, MXP_MAN_LEU0.5_SPD, MXP_MAN_LEU1_SPD).

The PXRD patterns of the physical mixtures (PMs) showed that MX, MXP, MAN, and LEU had crystalline structures. Since POL lacked crystalline characteristics, its presence had negligible effect on the diffractograms. The intensities of distinctive peaks declined in the case of SPD products (Figure 2 and Figure 3). Overall, the crystallinity of the APIs was reduced by the spray drying processes. The original MX and MXP exhibited a crystalline structure, and characteristic peaks of the formulations were decreased when compared to the PM. The API became amorphous following wet milling and spray drying, which may have aided in the dissolution process especially in the case of the MX-containing product. However, this amorphization may affect the stability of the final DPI.

### 2.5. Evaluation of Fourier-Transform Infrared Spectroscopy Investigation

**Figure 4:**
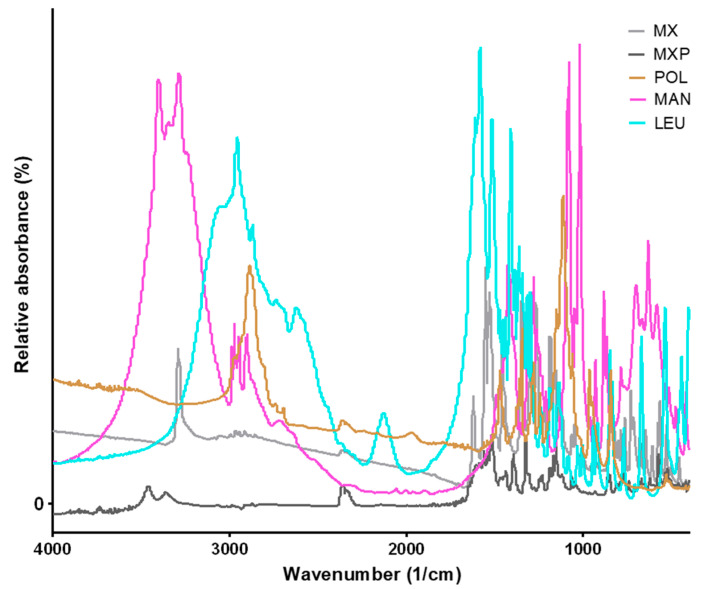
FTIR results of raw materials (MX, MXP, POL, MAN, LEU).

**Figure 5:**
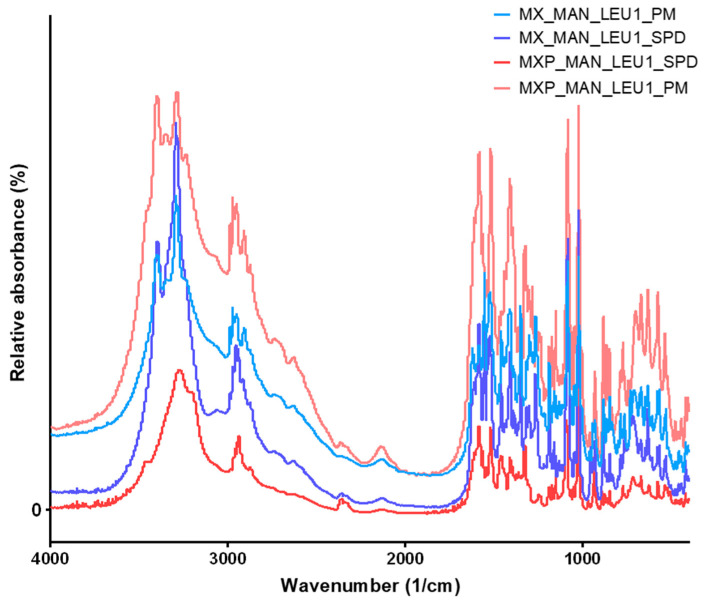
FTIR results of PMs (MX_MAN_LEU1_PM, MXP_MAN_LEU1_PM) and SPD samples (MX_MAN_LEU1_SPD, MXP_MAN_LEU1_SPD).

The Fourier-transform infrared spectroscopy (FT-IR) analysis was conducted to study the possible molecular interactions between MX, MXP, and the excipients (POL, MAN, LEU). To identify the changes occurring during the preparation methods, the FT-IR spectra of the original materials were recorded (Figure 4). After that, the highest LEU-containing SPD products were compared to the PMs. The most characteristic vibrations of MX were observed at 3290.2 cm^−1^ (N-H stretching), 1550.0 cm^−1^ (C=N stretching), 1456.7 cm^−1^ (C=C stretching of the aromatic ring), and 1346.5 cm^−1^ (S=O stretching vibrations of the sulfonyl groups) [48]. These peaks are observable in the case of the PMs (Figure 5). All the mentioned peaks of MX were detected in the SPD samples, indicating that the preparation process did not cause any structural changes. The peak of LEU (2950.4 cm^−1^) and MAN (3289.8 cm^−1^) did not change significantly during the preparation method. There was no considerable difference observed in the characteristic peaks in the FT-IR spectra of SPD formulations compared to pure drugs. This indicated that there was no interaction between drugs and excipients.

### 2.6. Outcomes of In Vitro Dissolution Investigation

**Figure 6:**
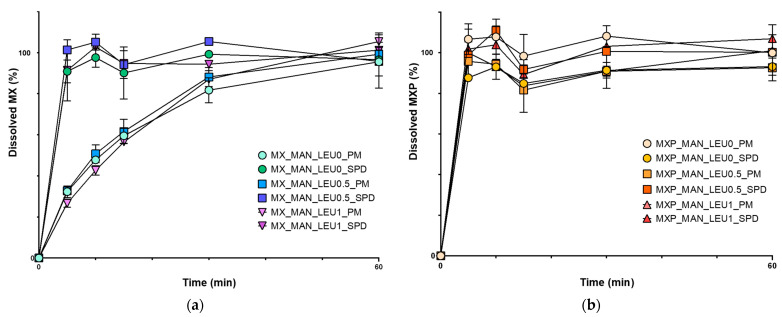
The results of the in vitro dissolution tests. (**a**) MX_MAN_LEU0_PM, MX_MAN_LEU0_SPD, MX_MAN_LEU0.5_PM, MX_MAN_LEU0.5_SPD, MX_MAN_LEU1_PM, MX_MAN_LEU1_SPD; (**b**) MXP_MAN_LEU0_PM, MXP_MAN_LEU0_SPD, MXP_MAN_LEU0.5_PM, MXP_MAN_LEU0.5_SPD, MXP_MAN_LEU1_PM, MXP_MAN_LEU1_SPD. Data are means ± SD (*n* = 3 independent measurements).

Due to the limited water solubility of MX, the samples which include raw materials had the lowest amount of MX released (Figure 6). All the MX was released from the SPD samples during the first five minutes. When compared to the PMs, the SPD samples demonstrated significantly improved drug release (MX_MAN_LEU0_PM, MX_MAN_LEU0_SPD: *p* < 0.0001; MX_MAN_LEU0.5_PM, MX_MAN_LEU0.5_SPD: *p* < 0.0001, MX_MAN_LEU1_PM, MX_MAN_LEU1_SPD: *p* < 0.001). These enhancements may be linked to the partial amorphization and increased specific surface area. The dissolution efficiency (DE%) values were the following: MX_MAN_LEU0_PM: 61.75 ± 2.67, MX_MAN_LEU0_SPD: 92.45 ± 4.26; MX_MAN_LEU0.5_PM: 64.79 ± 3.40, MX_MAN_LEU0.5_SPD: 97.77 ± 4.06, MX_MAN_LEU1_PM: 62.01 ± 2.59, MX_MAN_LEU1_SPD: 94.41 ± 2.08. Because of the high water solubility of MXP, the results for the MXP-containing SPD samples were comparable to the PMs. The DE% values were the following: MXP_MAN_LEU0_PM: 97.97 ± 15.14, MXP_MAN_LEU0_SPD: 80.40 ± 4.4; MXP_MAN_LEU0.5_PM: 81.30 ± 0.13, MXP_MAN_LEU0.5_SPD: 89,80 ± 0.41, MXP_MAN_LEU1_PM: 79.56 ± 11.926, MXP_MAN_LEU1_SPD: 90.20 ± 1.89.

### 2.7. Determined In Vitro Aerodynamic Properties of DPI Formulation

**Figure 7:**
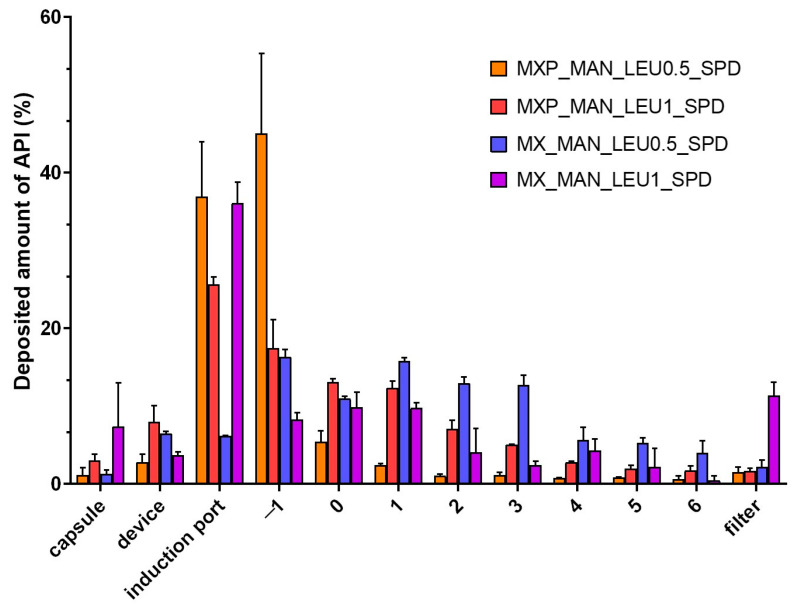
In vitro aerodynamic distribution of SPD samples (MX_MAN_LEU0.5_SPD, MX_MAN_LEU1_SPD, MXP_MAN_LEU0.5_SPD, MXP_MAN_LEU1_SPD). Data are means ± SD (*n* = 3 independent measurements).

According to the outcomes of the laser diffraction analysis, the LEU-containing formulations were determined during the aerodynamic assessment as they showed a particle size under 5 µm (except MX_MAN_LEU0.5_SPD). Figure 7 demonstrated the deposition of the samples across different Andersen cascade impactor (ACI) sections. The results of both preparations indicated adhesion at all levels but in varying amounts. We can draw the conclusion that the precise deposition is primarily determined by the API and cannot be reliably predicted based solely on the excipient system. The evaluated in vitro aerodynamic results by Inhalytix™ 2.0.6. software are presented in Table 3. The mass median aerodynamic diameter (MMAD) values were 4.42 and 4.68 µm in the case of MX-containing DPIs, which are in the required size range for pulmonary delivery. The FPF result was the best (52%) in the case of MX_MAN_LEU0.5_SPD, which is larger than the FPF values of the commercially available DPI formulations in the Breezhaler^®^ device (30%) [49]. When the two different API-containing samples were tested, a significant difference was calculated between the FPF values of MX_MAN_LEU0.5_SPD and MXP_MAN_LEU0.5_SPD (*p* < 0.001) and MX_MAN_LEU1_SPD and MXP_MAN_LEU01_SPD (*p* < 0.001). Emitted fraction (EF) was around 90% in all cases without any significant differences.

### 2.8. Results of Aerodynamic Characterization by Spraytec^®^ Device

Formulations containing LEU have undergone additional analysis using a Spraytec^®^ instrument. The ACI measurements and the aerodynamic diameter values were comparable (Table 4). D[0.5] values of MX_MAN_LEU0.5_SPD, MXP_MAN_LEU0.5_SPD, MX_MAN_LEU1_SPD, and MXP_MAN_LEU01_SPD showed significant difference (*p* < 0.001 and *p* < 0.05, respectively). However, only the MX_MAN_LEU0.5_SPD sample was smaller than 5 µm; for inhalation delivery, that composition would be optimal. The particle size results are larger than those of previous laser diffraction experiments. The explanation could relate to the agglomeration of particles during aerosolization.

## 3. Discussion

Overall, this study successfully demonstrated the applicability and versatility of a previously developed innovative excipient system by evaluating its performance with two APIs, MX and MXP. Both formulations underwent a comprehensive physicochemical characterization and dosage form evaluation, which provided a thorough understanding of their suitability for pulmonary drug delivery via DPIs.

In the case of the MX_MAN_LEU0.5_SPD and MX_MAN_LEU1_SPD samples, we successfully achieved a particle size under 5 μm (3.03 ± 0.10 and 3.36 ± 0.07 μm) according to laser diffraction measurement with a narrow PSD (1.46 ± 0.18 and 1.55 ± 0.14). This is a critical size range for optimal deposition in the lower respiratory tract while avoiding deposition in the oropharyngeal region, thereby maximizing therapeutic efficacy [50]. In the case of MX- and MXP-containing samples, the morphological analysis of the formulations revealed that the particles exhibited a predominantly spherical shape. This morphology is favorable for inhalation powders, as it often correlates with improved flowability and aerodynamic behavior, both key parameters for consistent dose delivery [14]. The outcomes of the density measurement (tapped density ~0.3 g/cm^3^) also predicted an improved aerodynamic performance. Regarding the structural analysis, partial amorphization was observable, which influenced the dissolution profile, and it may affect the stability of the formulation. FTIR showed that there were no molecular interactions between the components after the preparation procedure. One of the most promising results was the dissolution profile of the MX-containing samples, which demonstrated complete drug release within 5 min. Such rapid dissolution is particularly advantageous for pulmonary administration, where a fast onset of action may be desired. The dissolution behavior also reflects the effectiveness of the excipient matrix in enhancing the wettability and solubility of the poorly water-soluble MX. The aerodynamic properties of the formulations further supported their potential for inhalation therapy. MX_MAN_LEU0.5_SPD showed the best performance, with MMAD value being 4.42 ± 0.07 μm, aligning well with the targeted deposition zone within the lungs. The FPF value exceeded 50% (52.10 ± 0.71%), which predicts the efficiency of the powder to reach the peripheral lung regions. EF was outstanding: 91.86 ± 0.50% of the sample was released from the device. These characteristics confirm that the formulation meets the criteria for effective pulmonary delivery.

Although certain formulation characteristics—such as dissolution rate and aerosol performance—were influenced by the specific physicochemical properties of the API used, our findings clearly demonstrate that the novel excipient system and production protocol can be reliably adapted to accommodate different APIs. Among the various formulations tested, MX_MAN_LEU0.5_SPD exhibited the most favorable overall performance. This formulation, which employed a specific ratio of additives, can serve as a reference point for future DPI development and optimization efforts. Importantly, when compared to previous results obtained using ibuprofen as a model drug [46], the current findings further validate the robustness and flexibility of this excipient platform. It appears to be particularly well-suited for improving the delivery of poorly water-soluble drugs to the lungs, which remains a significant challenge in pulmonary therapeutics. In conclusion, the successful application of this excipient system to two new APIs suggests that it has strong potential for broader use in inhalation drug development. Future work should focus on scaling up the production process, evaluating long-term stability, and conducting in vivo performance studies. Ultimately, this strategy could contribute to more rapid and efficient development of DPI formulations, enabling improved patient outcomes across a range of respiratory and systemic conditions.

## 4. Materials and Methods

### 4.1. Materials

The APIs were meloxicam (MX) and meloxicam-potassium monohydrate (MXP) (Egis Pharmaceuticals PLC., Budapest, Hungary). Poloxamer 188 (POL) (Sigma-Aldrich Co. Ltd., Budapest, Hungary), D-Mannitol (MAN) (Molar Chemicals Kft, Halásztelek, Hungary), and L-leucine (LEU) (AppliChem GmbH, Darmstadt, Germany) were chosen as excipients.

### 4.2. Methods

#### 4.2.1. Preparation of Solution of MXP and the Pre-Suspension of MX

The water-soluble MXP-containing formulation was prepared by dissolving MXP, POL, MAN, and LEU in purified water in different concentrations (Table 5). In the case of the MX-containing formulation, POL was solved in purified water, which resulted in a solution with a 1.0% (*w*/*w*%) concentration. POL prevents the aggregation of the MX particles during the grinding method. It was followed by the preparation of a suspension, which contained 2.00 g of raw MX and 18.0 g of 1.0% POL solution. The milling was performed with 20.00 g of ZrO_2_ beads in a high-performance planetary ball mill (Fritsch Planetary Micro Mill Pulverisette 7, Fritsch GmbH, Idar-Oberstein, Germany). The milling process was modified to reach a microsuspension similar to the previously developed ibuprofen microsuspension. The parameters were the following: rotation speed: 400 rpm; milling time: 10 min [41,46,48].

#### 4.2.2. Spray Drying of the Solution of MXP and the Pre-Suspension of MX

DPI systems were spray-dried (SPD), using the same parameters for both preparations. Three different formulations were produced from the MXP solution, and three different compositions were formulated from the MX suspension by adding MAN and various amounts of LEU. The composition of the final formulations is shown in Table 5. LEU enhances the dispersity of the SPD powders during aerosolization. The solid dosage forms were produced with a Büchi Mini Spray Dryer (Büchi Mini Spray Dryer B-191, Büchi, Flawil, Switzerland). Based on our previous experiments, the spray drying settings were the following: inlet temperature: 70 °C; aspirator capacity: 85%; airflow rate: 500 L/h; pump rate: 10% [42,46].

#### 4.2.3. Preparation of Physical Mixtures

From the initial materials, three physical mixtures (PMs) were created. They shared the same compositions as the samples that were dried (Table 5). The characteristics of the PMs were examined in comparison with those of the spray-dried products during the investigations.

#### 4.2.4. Determination of API Content

The API content (Table 2) of DPIs was determined by solving 1.0 mg of powder in 25 mL of pH 7.4 phosphate buffer in the case of MXP and in 25 mL of a mixture of methanol and pH 7.4 phosphate buffer (90 + 10 *v/v%*) in the case of MX. The solutions were analyzed by UV/Vis spectrophotometry (Jasco V-730 UV/VIS Spectrophotometer, Jasco Inc., Easton, MD, USA) at a wavelength of 362 nm (Table 5).

#### 4.2.5. Laser Diffraction-Based Particle Size Measurement

Laser diffraction was used to determine the particle size, the particle size distribution (PSD), and the specific surface area (SSA) of our samples (Malvern Mastersizer Scirocco 2000, Malvern Instruments Ltd., Worcestershire, UK). The refractive index was adjusted to 1.72. The wet dispersion unit was used to analyze the particle size of the MX suspension. The suspension was measured in purified water with stirring at 2000 rpm. The dry dispersion unit was used to test the SPD powders. The air pressure was set to 3.0 bar, and 75% vibration feed was used. The PSD was characterized by the values of D[0.1] (10% of the volume distribution is below this value), D[0.5] (50% of the volume distribution is below this value), and D[0.9] (90% of the volume distribution is below this value). Span values were calculated based on Equation (1). The SSA was derived from the PSD data. The calculations were made under the assumption of spherical particles.

(1) $$Span=\frac{D\left[0.9\right]-D[0.1]}{D[0.5]}$$

#### 4.2.6. Scanning Electron Microscopy Investigation

Scanning electron microscopy (SEM) (Hitachi S4700, Hitachi Scientific Ltd., Tokyo, Japan) was used to investigate the morphology of the SPD samples. The conditions were the following: 10 kV high voltage, 10 mA amperage, and 1.3–13.1 mPa air pressure. A high vacuum evaporator and argon atmosphere were used to make the sputter-coated samples conductive with gold–palladium (Bio-Rad SC 502, VG Microtech, Uckfield, UK).

#### 4.2.7. Density and Powder Flow Measurement

The densities of the formulations were measured using a tap density tester (ETD-1020x, Electrolab, Mumbai, India). A cylinder was filled with 1.5–2.0 cm^3^ of powders to calculate the bulk density (ρ_b_). It was tapped 1000 times [51]. The tapped density (ρ_t_) was calculated compared to the volume of the powder before and after the taps. The Hausner ratio (HR) and Carr index (CI) values of the samples were evaluated from the ρ_b_ and the ρ_t_ (Equations (2) and (3)).

(2) $$\mathrm{HR}=\frac{\rho_{t}}{\rho_{b}}$$

(3) $$\mathrm{CI}=\frac{\left(\rho_{t}-\rho_{b}\right)}{\rho_{t}}*100\;$$

#### 4.2.8. Powder X-Ray Diffraction Analysis

Powder X-ray diffraction (PXRD) spectra were recorded with the help of the BRUKER D8 Advance X-ray diffractometer (Bruker AXS GmbH, Karlsruhe, Germany) to analyze the crystalline structure of the samples. The radiation source was Cu Kλ1 radiation (λ = 1.5406 Å). The parameters were the following: Cu target, Ni filter, 40 kV voltage, 40 mA current, time constant 0.1°/min, angular step 0.010° over the interval of 3–40°. DIFFRACT plus EVA 28 software (Bruker AXS GmbH, Karlsruhe, Germany) was applied for the evaluation.

#### 4.2.9. Fourier-Transform Infrared Spectroscopy Investigation

The interactions between the materials were investigated by Fourier-Transform Infrared Spectroscopy (FT-IR), using the AVATAR 330 FT-IR spectrometer (Thermo Nicolet, Thermo Fisher Scientific Inc., Waltham, MA, USA). The powders were homogenized with 150 mg of KBr in an agate mortar, and the mixtures were pressed to prepare pastilles using a Specac^®^ hydraulic press (Specac, Inc., Orpington, UK) with 10-ton pressing force. The spectra were recorded from 4000 to 400 cm^−1^ at an optical resolution of 4 cm^−1^.

#### 4.2.10. In Vitro Dissolution Test in Simulated Lung Media

A modified paddle method (Hanson SR8 Plus, Teledyne Hanson Research, Chatsworth, Los Angeles, CA, USA) from the European Pharmacopeia was used to define the release of MX and MXP from formulations [52]. There are no available regulatory requirements for in vitro dissolution testing of inhaled powders. Moreover, the exact volume of the lung lining fluid cannot be accurately determined. The estimated value is between 10 and 70 mL [53,54]. In our case, 50 mL of the simulated lung media were applied during the measurement. The samples contained 1.5 mg of MX and MXP, which is a tenth of the highest oral dose of MX [31] and the estimated dose of MX for pulmonary delivery. The paddle was rotated at 100 rpm for continuous homogenization. The measurement was performed for 60 min at 37 °C. A total of 5 mL of the samples was taken out after 5, 10, 15, 30, and 60 min. The medium was replenished after every sampling. After filtration (pore size: 0.45 µm, Millex-HV syringe-driven filter unit, Millipore Corporation, Bedford, MA, USA), the dissolved quantity of MX and MXP was determined spectrophotometrically at a wavelength of 362 nm (Jasco V-730 UV/VIS Spectrophotometer, Jasco Inc., Easton, MD, USA).

#### 4.2.11. In Vitro Aerodynamic Measurements

The aerosolization behavior of the SPD formulations were analyzed in vitro, using an Andersen cascade impactor (ACI) (Apparatus D, Copley Scientific Ltd., Nottingham, UK) [55]. The inhalation flow rate was set to 60 L/min (High-capacity Pump Model HCP5, Critical Flow Controller Model TPK, Copley Scientific Ltd., Nottingham, UK). The flow rate through the impactor was measured by a mass flow meter (Flow Meter Model DFM 2000, Copley Scientific Ltd., Nottingham, UK). The inhalation time was set to 4 s. The setting models the normal breathing pattern with a 4 L inhalation volume. Breezhaler^®^ single-dose devices (Novartis International AG, Basel, Switzerland) were used, with transparent, size 3 hydroxypropyl methylcellulose capsules (Ezeeflo™, ACG-Associated Capsules Pvt. Ltd., Mumbai, India). The collection plates on the stages were coated with a Span 85 and cyclohexane (1 + 99 *w*/*w*%) mixture to mimic the pulmonary adhesive circumstances. The device, the capsules, the induction port, the plates, and the filter were washed with methanol and pH 7.4 phosphate buffer (10 + 90 *v/v%)* to collect and dissolve the deposited amount of MX. In the case of MXP-containing samples, the parts of the ACI were washed with pH 7.4 phosphate buffer. The API was quantified by UV/Vis spectrophotometry (Jasco V-730 UV/VIS Spectrophotometer, Cambridge, UK) at a wavelength of 362 nm. The in vitro aerodynamic properties were calculated by Inhalytix™ 2.0.6. (Copley Scientific Ltd., Nottingham, UK) software, which is a validated aerodynamic particle size distribution data analysis program. Fine particle fraction (FPF), emitted fraction (EF), and median mass aerodynamic diameter (MMAD) were determined. FPF is defined as the percentage of the mass of the API consisting of particles with an aerodynamic diameter of fewer than 5 μm divided by the emitted dose of the formulations. MMAD is influenced by the inhalation flow rate, density, size, and shape of the particle. EF is the released fraction from the DPI device.

#### 4.2.12. Aerodynamic Particle Size Analysis Using Spraytec^®^ Device

The aerodynamic diameter was also determined using a Spraytec^®^ laser diffractometer equipped with an inhalation cell (Malvern Instruments Ltd., Worcestershire, UK) and ACI. The device measures PSD directly from the inhalation device. SPD formulations were aerosolized from 3 cellulose capsules loaded into a Breezhaler^®^ device connected to the inhalation cell through an induction port. By connecting the assembly to an ACI, a closed system was established that permitted size measuring under carefully monitored conditions [56]. The inhalation flow rate was set at 60 L/min. The inhalation time was 4 s.

#### 4.2.13. Statistical Analysis

Statistical analysis was performed by Welch’s *t*-test using GraphPad Prism 8.0.1. software (GraphPad Software, La Jolla, CA, USA). *p*-values < 0.05 indicated statistically significant differences.

## 5. Conclusions

This study confirmed the effectiveness and adaptability of a novel excipient system for DPI formulations by successfully applying it to two distinct APIs, MX and MXP. The comprehensive physicochemical and aerodynamic characterization revealed that the formulations, particularly MX_MAN_LEU0.5_SPD, met the critical criteria for efficient pulmonary delivery, including optimal particle size distribution, favorable morphology, rapid dissolution, and high fine particle fraction. Furthermore, the consistency of these results across different APIs highlights the robustness and versatility of the excipient formula. These findings not only reinforce the potential of this system for broad application in inhalation drug development but also lay a solid foundation for future work focused on scale-up, stability assessment, and in vivo evaluation.

## Figures and Tables

**Table 1:** Particle size of spray-dried (SPD) samples. Data are means ± SD (*n* = 3 independent measurements).

Sample	D[0.1] (μm)	D[0.5] (μm)	D[0.9] (μm)	Span	SSA (m^2^/g)
MX_MAN_LEU0_SPD	1.90 ± 0.35	4.53 ± 0.10	16.29 ± 3.47	3.18 ± 0.70	1.87 ± 0.33
MX_MAN_LEU0.5_SPD	1.43 ± 0.10	3.03 ± 0.10	5.85 ± 0.58	1.46 ± 0.18	2.37 ± 0.08
MX_MAN_LEU1_SPD	1.54 ± 0.10	3.36 ± 0.07	6.77 ± 0.45	1.55 ± 0.14	2.16 ± 0.07
MXP_MAN_LEU0_SPD	4.63 ± 2.10	18.92 ± 14.31	54.31 ± 14.86	3.26 ± 1.27	0.79 ± 0.29
MXP_MAN_LEU0.5_SPD	4.05 ± 0.52	17.04 ± 0.61	86.78 ± 17.51	4.88 ± 1.23	0.73 ± 0.10
MXP_MAN_LEU1_SPD	1.68 ± 0.10	3.90 ± 0.80	18.85 ± 12.42	4.09 ± 2.46	1.86 ± 0.33

**Table 2:** Density results of SPD samples. Data are means ± SD (*n* = 3 independent measurements).

Sample	Bulk Density (g/cm^3^)	Tapped Density (g/cm^3^)	Hausner Ratio	Carr Index
MX_MAN_LEU0_SPD	0.23 ± 0.03	0.36 ± 0.01	1.61 ± 0.25	36.90 ± 9.22
MX_MAN_LEU0.5_SPD	0.26 ± 0.03	0.38 ± 0.03	1.48 ± 0.19	31.64 ± 8.16
MX_MAN_LEU1_SPD	0.21 ± 0.02	0.34 ± 0.06	1.63 ± 0.22	37.91 ± 7.92
MXP_MAN_LEU0_SPD	0.12 ± 0.00	0.19 ± 0.01	1.61 ± 0.10	37.78 ± 3.85
MXP_MAN_LEU0.5_SPD	0.20 ± 0.04	0.32 ± 0.05	1.56 ± 0.05	35.73 ± 2.15
MXP_MAN_LEU1_SPD	0.16 ± 0.00	0.27 ± 0.01	1.64 ± 0.07	39.20 ± 2.41

**Table 3:** In vitro aerodynamic properties of SPD samples according to ACI: MMAD, FPF, and EF. Data are means ± SD (*n* = 2 independent measurements).

Sample	MMAD (μm)	FPF (%)	EF (%)
MX_MAN_LEU0.5_SPD	4.42 ± 0.07	52.10 ± 0.71	91.86 ± 0.50
MX_MAN_LEU1_SPD	4.68 ± 0.19	31.73 ± 1.78	88.92 ± 5.10
MXP_MAN_LEU0.5_SPD	6.28 ± 0.51	26.87 ± 2.03	87.84 ± 2.03
MXP_MAN_LEU1_SPD	-	7.92 ± 3.07	95.68 ± 0.25

**Table 4:** In vitro aerodynamic properties determined by Spraytec^®^ device of SPD samples. Data are means ± SD (*n* = 3 independent measurements).

Sample	D[0.5] (μm)	Span	SSA (m^2^/g)
MX_MAN_LEU0.5_SPD	4.58 ± 0.15	2.52 ± 0.16	4.23 ± 0.14
MX_MAN_LEU1_SPD	5.72 ± 0.05	2.40 ± 0.16	3.10 ± 0.10
MXP_MAN_LEU0.5_SPD	39.62 ± 1.73	3.92 ± 0.62	0.48 ± 0.03
MXP_MAN_LEU1_SPD	8.30 ± 0.56	4.04 ± 0.44	2.13 ± 0.15

**Table 5:** Composition of SPD and PM samples. Data are means ± SD (*n* = 3 independent measurements).

Sample	MX (g)	MXP (g)	POL (g)	MAN (g)	LEU (g)	API Content (%)
MX_MAN_LEU0_PM	1.00	-	0.09	2.00	0.00	32.36
MX_MAN_LEU0.5_PM	1.00	-	0.09	2.00	0.50	27.86
MX_MAN_LEU1_PM	1.00	-	0.09	2.00	1.00	24.45
MX_MAN_LEU0_SPD	1.00	-	0.09	2.00	0.00	30.50 ± 0.59
MX_MAN_LEU0.5_SPD	1.00	-	0.09	2.00	0.50	25.60 ± 0.77
MX_MAN_LEU1_SPD	1.00	-	0.09	2.00	1.00	22.77 ± 0.29
MXP_MAN_LEU0_PM	-	1.00	0.09	2.00	0.00	32.36
MXP_MAN_LEU0.5_PM	-	1.00	0.09	2.00	0.50	27.86
MXP_MAN_LEU1_PM	-	1.00	0.09	2.00	1.00	24.45
MXP_MAN_LEU0_SPD	-	1.00	0.09	2.00	0.00	29.43 ± 1.15
MXP_MAN_LEU0.5_SPD	-	1.00	0.09	2.00	0.50	24.07 ± 1.06
MXP_MAN_LEU1_SPD	-	1.00	0.09	2.00	1.00	21.52 ± 0.61

## Data Availability

The original contributions presented in the study are included in the article; further inquiries can be directed to the corresponding author.

## Abbreviations

The following abbreviations are used in this manuscript:

ACI	Andersen cascade impactor
API	Active pharmaceutical ingredient
CI	Carr index
D[0.1]	10% of the volume distribution is below this value
D[0.5]	50% of the volume distribution is below this value
D[0.9]	90% of the volume distribution is below this value
DE%	Dissolution efficiency
DPI	Dry powder inhaler
EF	Emitted fraction
FPF	Fine particle fraction
FT-IR	Fourier-transform infrared spectroscopy
HR	Hausner ratio
LEU	L-leucine
MAN	Mannitol
MMAD	Mass median aerodynamic diameter
MX	Meloxicam
MXP	Meloxicam-potassium monohydrate
NSAID	Non-steroidal anti-inflammatory drug
PM	Physical mixture
POL	Poloxamer-188
PSD	Particle size distribution
SD	Standard deviation
SEM	Scanning electron microscopy
SPD	Spray-dried
PXRD	Powder X-ray diffraction
ρ_b_	Bulk density
ρ_t_	Tapped density
